# Genome sequencing of *Aspergillus glaucus* ‘CCHA’ provides insights into salt-stress adaptation

**DOI:** 10.7717/peerj.8609

**Published:** 2020-02-24

**Authors:** Wenmin Qiu, Jingen Li, Yi Wei, Feiyu Fan, Jing Jiang, Mingying Liu, Xiaojiao Han, Chaoguang Tian, Shihong Zhang, Renying Zhuo

**Affiliations:** 1 State Key Laboratory of Tree Genetics and Breeding, Chinese Academy of Forestry, Beijing, China; 2 The Research Institute of Subtropical Forestry, Chinese Academy of Forestry, Hangzhou, Zhejiang, China; 3 Key Laboratory of Systems Microbial Biotechnology, Tianjin Institute of Industrial Biotechnology, Chinese Academy of Sciences, Tianjin, China; 4 College of Plant Sciences, Jilin University, Changchun, China

**Keywords:** Genome, Transcriptome, *Aspergillus glaucus*, ‘CCHA’, Salt-stress-related genes, Transgenic arabidopsis

## Abstract

*Aspergillus*, as a genus of filamentous fungi, has members that display a variety of different behavioural strategies, which are affected by various environmental factors. The decoded genomic sequences of many species vary greatly in their evolutionary similarities, encouraging studies on the functions and evolution of the *Aspergillus* genome in complex natural environments. Here, we present the 26 Mb de novo assembled high-quality reference genome of *Aspergillus glaucus* ‘China Changchun halophilic *Aspergillus*’ (CCHA), which was isolated from the surface of plants growing near a salt mine in Jilin, China, based on data from whole-genome shotgun sequencing using Illumina Solexa technology. The sequence, coupled with data from comprehensive transcriptomic survey analyses, indicated that the redox state and transmembrane transport might be critical molecular mechanisms for the adaptation of *A. glaucus* ‘CCHA’ to the high-salt environment of the saltern. The isolation of salt tolerance-related genes, such as *CCHA-2114*, and their overexpression in *Escherichia coli* demonstrated that *A. glucus* ‘CCHA’ is an excellent organism for the isolation and identification of salt tolerant-related genes. These data expand our understanding of the evolution and functions of fungal and microbial genomes, and offer multiple target genes for crop salt-tolerance improvement through genetic engineering.

## Introduction

*Aspergillus* species exhibit amazingly diverse behavioural and physiological characteristics, as well as varied capacities to adapt to extreme environments. Measures for managing diverse environmental stresses have evolved in different *Aspergillus* species. Therefore, they are considered to be good materials for studying adaptation and responses to many natural and man-made environmental stressors (Gibbons & Rokas, 2013). Large-scale genomic projects in several *Aspergillus* members have provided insights into the genetic and molecular mechanisms involved in environmental stress responses (De Vries et al., 2017; Linz, Wee & Roze, 2014; Pereira Silva et al., 2016). However, to further clarify the conserved characteristics of the adaptive responses of *Aspergillus* to specific stresses, and to trace the evolutionary processes involved in its environmental adaptation and responses, insights into *Aspergillus* species in different evolutionary locations, such as *Aspergillus glaucus*. *A. glaucus* occupies diverse fungal niches, with varied environmental stresses, and thus offers a large number of adaptation and stress-response models (Abrashev et al., 2016; Liu et al., 2016; Takenaka et al., 2019). As a fungus that commonly appears in the environment, associations of *A. glaucus* with extremophilic microbes habe been rarely described. A strain of the species, *A. glaucus* ‘China Changchun halophilic *Aspergillus*’ (CCHA) was isolated from the surface of wild vegetation growing around a saltern in Jilin, China (Liu et al., 2011), which indicated that it may have a unique salt-stress resistance mechanism. Even though this strain was discovered over 10 years ago, the lack of genetic information has limited our understanding of its salt tolerance. To study the molecular mechanisms underlying the salt tolerance of *A. glaucus* ‘CCHA’, a high-quality genome draft was required.

Extremophiles, which grow in harsh conditions, like hypersaline environments, not only survive, but flourish, in environments that are hostile to other creatures (Mesbah & Wiegel, 2012). Microbes adapt to high-salt concentrations by developing special strategies. Halotolerant fungi counters salt stress not by accumulating internal high ion amounts as seen in halophilic Archaea, but by morphological adaption (Zajc et al., 2013), strengthening cell walls, accumulating osmolytes, such as glycerol, and modifying genetic structures (Duran, Cary & Calvo, 2010; Kralj et al., 2010; Kis-Papo et al., 2014). Changes resulting from salt stress at the gene expression level are intricate, and the underlying molecular mechanisms are not well known. RNA-seq is a method for discovering, profiling, and quantifying RNA transcripts to provide valuable information for understanding of gene functions, cell responses and evolution. Adaptive responses mediated by differentially expressed genes (DEGs) analyses have been analysed to increase our understanding of the transcript expression levels in various *Aspergillus* species, such as *Aspergillus flavus*, *Aspergillus niger* and *Aspergillus fumigatus* (Lv et al., 2018; Gomaa et al., 2017; Liu et al., 2011; Takahashi et al., 2017) using RNA-seq over the last several years. However, such investigations in *A. glaucus* have not been reported.

Here, we assembled a high-quality reference genome for *A. glaucus* ‘CCHA’ based on sequence data from whole-genome shotgun sequencing platform using Illumina Solexa technology. This assembled genome included 106 scaffolds (>1 KB; N50 = ~0.795 MB), has a length of ~26.0 MB and covers ~83% of the predicted genome size (~31.6 MB). Using data analyses of comprehensive transcriptomic surveys of the six different *A. glaucus* ‘CCHA’ treatment groups and comparative genomic analyses with other *A. glaucus* strains, we investigated the molecular mechanisms of the metabolic system’s evolution in *A. glaucus* fungal species and their adaptations to the high-salt environment of the saltern. They provided an excellent resource for the discovery of novel candidate gene for salt tolerance in fungi.

## Methods

### Microorganisms, culture medium and inoculum development

The sampling site for the isolation of salt-tolerant *A. glaucus* ‘CCHA’ was the air-dried surface of wild vegetation from a solar salt field in Northeast China. For fungal isolation, potato extract plus 20 g · L^−1^ glucose, supplemented with 250 g · L^−1^ NaCl was used. The production of an intense greyish-green colour under and around the fungal colony in the salt-supplemented agar was considered a halophilic positive reaction. The strain was identified based on morphological characterisations and subsequently confirmed by 5.8S rDNA sequencing of fungal genomes from NCBI. The strain was deposited under the accession name: CCHA. A defined medium, composed of 20 g · L^−1^ glucose and 200 g · L^−1^ potato extract prepared in 50 g · L^−1^ NaCl, was used for growth and maintenance. Fully sporulated slants were stored at 4 °C and subcultured once every 2 weeks. After being dislodged using a sterile inoculation loop under precise sterile conditions, the spores in the suspension were characterised by microscopy. The effects of salinity on the growth of the fungal isolate were determined by preparing the medium supplemented with NaCl to a final saline concentration of 1–8% (in 1% increases, e.g. 1 for 1%, 2 for 2% and 8 for 8%) and 9–21% (in 2% increases, e.g. 9 for 9%, 10 for 11% and 15 for 21%) for 6 days under 30 °C.

### Genome sequencing, assembly and validation

High-quality genomic DNA was extracted from the fruiting bodies using a modified Benzy method (Min et al., 1995). RNase A and proteinase K were separately used to remove RNA and protein contamination, respectively. The quantity and quality of the isolated DNA were separately checked by electrophoresis on a 1.0% agarose gel and on a Nanodrop 2000 spectrophotometer (Thermo Scientific, Waltham, MA, USA). Three Solexa genomic sequencing libraries (500 bp, 2 KB and 15 KB) were separately constructed using Cre-Lox recombination following the Illumina instructions (Van Nieuwerburgh et al., 2012) and subsequently sequenced using an Illumina HiSeq 2000 platform. Paired-end and mate-paired reads from Solexa sequencing were assembled by SOAPdenovo to construct contigs (Li et al., 2010). The contigs were assembled using the GS DE novo Assembler (Roche, 2011). A total of 2,595.2 M Illumina reads were selected to perform the genome size estimation. The distribution of 17-*kmer* had a major peak at 82x. Based on the total number *kmers* and the corresponding *kmer* depth of 84, the *A. glaucus* ‘CCHA’ genome size was estimated using the formula: Genome size = *kmer*_number/Peak_Depth.

### Genome annotation

Putative protein-coding genes of *A. glaucus* were predicted by combining several different ab initio gene predictors and sequence evidence, including protein sequences from closely related species and ESTs assembled in this study. The quality validation of gene models was evaluated by aligning the transcriptome, Ascomycota BUSCOs and homologous peptides to our gene predictions. A popular software tool RepeatMasker was used to identify and classify the repetitive elements (Tarailo-Graovac & Chen, 2009).

### Comparative genomic analysis

A comparative genomic analysis was performed using the MAUVE programme (Version 2.3.1). Default scoring and parameters were used to generate the alignment. Unique and shared gene families among *A. glaucus* ‘CCHA’ and other *Aspergillus* strains (AN513, AG516 and AA106) were clustered using the OrthoMCL method.

### Transcriptome sequencing and analysis

The strains of every sample were frozen in liquid nitrogen, and the total RNA was extracted using TRIzol and then treated with Dnase I according to the manufacturers’ protocols. mRNA purified by Sera-Mag magnetic oligo (dT) beads (Illumina, San Diego, CA, USA) from 20 µg total RNA per sample was reverse transcribed into double-stranded cDNA to generate the DNA libraries. The cDNA ends were repaired, and the cDNA was amplified, denatured and then sequenced on an Illumina Genome Analyzer IIx using special reagents.

The RNA-seq reads were aligned to the genome of *A. glaucus* ‘CCHA’ using the Hisat2 programmes (Trapnell, Pachter & Salzberg, 2009). Gene expression levels were measured in terms of FPKM values, for which the expression level of each gene is the sum of the values of its isoforms (Toung et al., 2011). The criteria FDR ≤ 0.05 and |log2FC| >= 1 were used to verify the significance of gene expression differences using edgeR.

### Isolation of salt-stress-related genes

Construction of a size-fractionated cDNA library was performed using the SMART cDNA Library Construction Kit (Clontech, Mountain View, CA, USA) according to the manufacturer’s instructions, with minor modifications (Fang et al., 2014). *E. coli* BL21 was transformed separately using cDNA obtained from plasmid pools and selected for salt stress on a 0.75-M NaCl-containing medium that led to the full repression of the empty vector. The resulting transformants were replicated onto media containing different salt concentrations and screened for strains that grew a rate similar to the empty vector-containing transformants on normal media but grew more readily on the salt-stress media.

### Phenotypic analysis of transgenic *Arabidopsis thaliana*

The recombinant pBI121 vector, into which *CCHA-2114* cDNA was inserted, was transformed into *A. thaliana* (Columbia ecotype) plants by *Agrobacterium tumefaciens* ‘EHA105’. Wild type (WT) and T3-homozygous CCHA-2114-overexpressing *A. thaliana* plants were sown in soil under long-day conditions (16 h light, 8 h dark). After 30 days, transgenic plants and control plants were soaked in Hoagland’s medium for 1, 3 and 5 days with or without 200 mm NaCl. Total SOD activity and MDA levels, a biomarker for lipid peroxidation, were assayed using previously described methods (Giannopolitis & Ries, 1977; Li et al., 2017). The degree of ion leakage was calculated as follows: conductivity (before boiling)/conductivity (after boiling) × 100%. The total chlorophyll, chlorophyll a and chlorophyll b contents were measured after extraction with 80% acetone (Fang et al., 2014). All the experiments were conducted at least three times independently in four lines (WT and three transgenic plant lines).

## Results

### Strain isolation and the salt-tolerance assessment of *A. glaucus* ‘CCHA’

**Figure 1:**
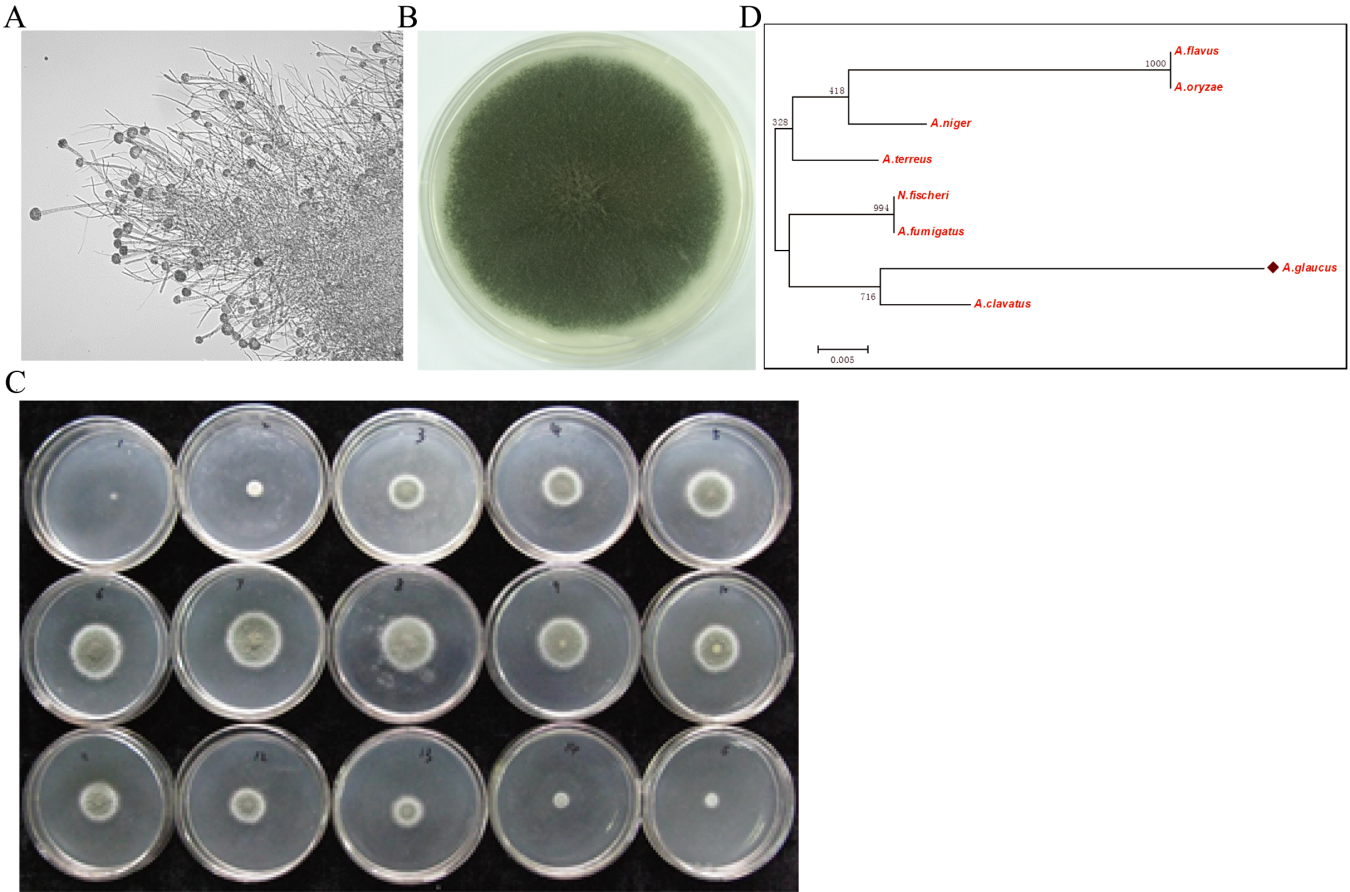
Isolation of *A. glaucus* ‘CCHA’ strain and salt-tolerance tests. (A) Micrographs of CCHA strain; (B) Appearance of CCHA strain in plate; (C) The salt-tolerance tests of CCHA strain. The concentration of NaCl is 1%–8% (Salt concentration increases in 1% increments), 9–15: the concentration of NaCl is 9%–21% (Salt concentration increases in 2% increments); (D) Phylogenetic tree analysis of *A. glaucus* and other *Aspergillus strains*.

**Figure 2:**
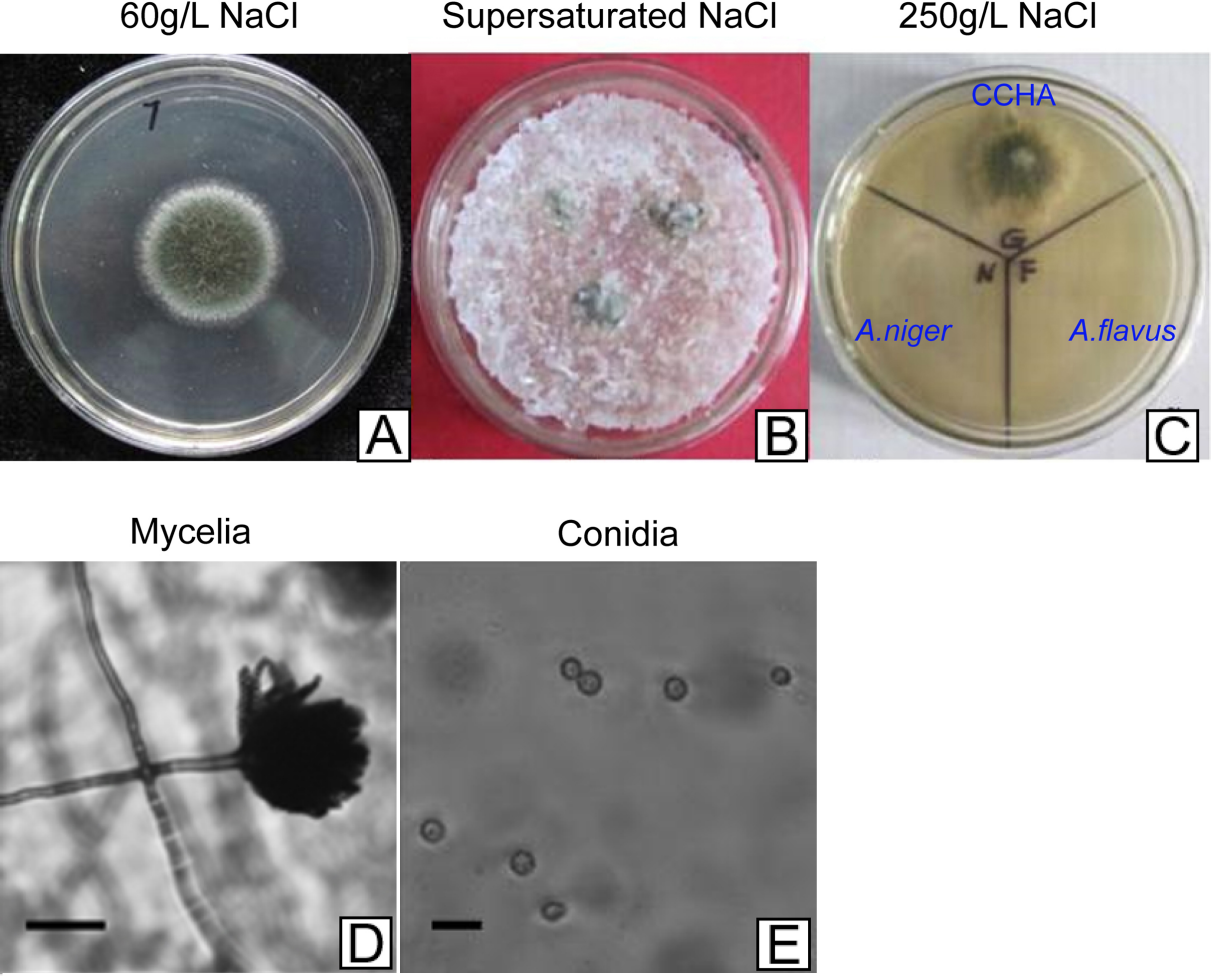
Development of *A. glaucus* ‘CCHA’ under different salt treatment conditions. (A–C) Solid plate cultures under different salt-treatment conditions, the salt concentration on the supersaturated is 500 g/L. (D) and (E) Appearance of mycelia and conidia observed by microscope.

The halophilic fungal strain was isolated from the surface of wild vegetation growing around a saltern, and it was identified as *A. glaucus* strain CCHA based on morphological properties, an ITS sequence comparison (Liu et al., 2011), and its salt-tolerance level (Figs. 1A–1C). The strain was extremely tolerant to salt levels over 25%, which is much greater than the levels tolerated by halotolerant *A. niger* and *A. flavus* (Figs. 1B and 2A–2E). The phylogenetic tree of the CCHA 5.8S rDNA sequence and the corresponding sequences of seven other reported *Aspergillus* strains provided insights into the evolution of the *A. glaucus* genome (Fig. 1D).

### Genome sequencing, assembly and annotation

**Figure 3:**
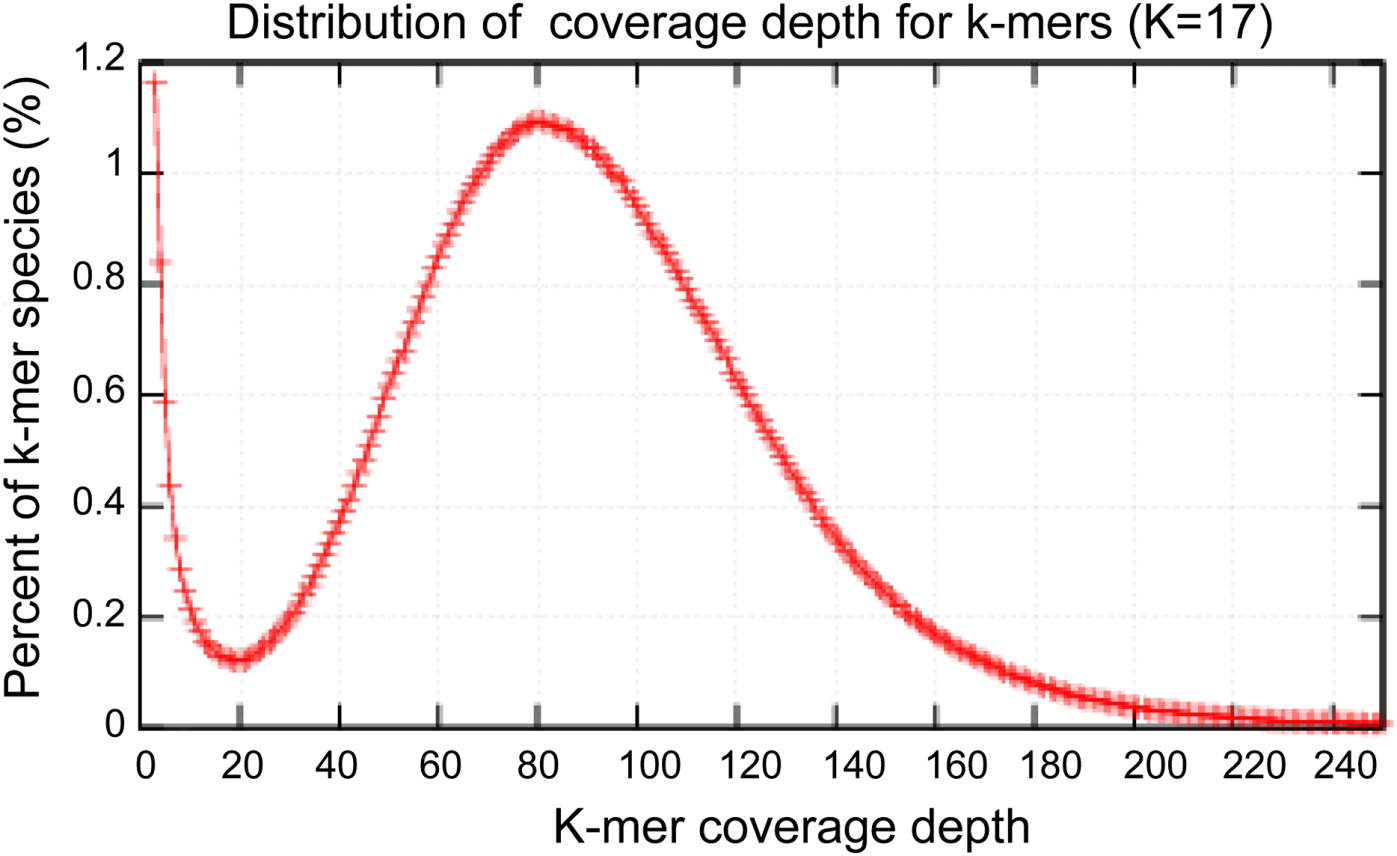
Estimation of the genome sizes based on k-mer statistics. Estimate the size of genome: 2,595.2M/82 = 31.6M.

Because microbial genomes adapt to environmental conditions (Chan et al., 2014; Gibbons & Rokas, 2013), to better use the RNA-seq data to confirm the candidate gene of saltern-isolated *A. glaucus* CCHA, we denovo sequenced the *A. glaucus* ‘CCHA’ genome using Illumina Solexa sequencing technology (Table 1). We generated approximately 17.5 GB of raw sequencing read data, resulting in a 358× genome coverage. The obtained reads were assembled using the Berry Genomics Inc. platform, which yielded a high-quality genome assembly. The N50 length of the contigs was 209 KB and the longest contig was 474 KB. The complete genome of *A. glaucus* ‘CCHA’ contained 76 chromosomal scaffolds of approximately 26 MB (82% coverage of the 31.6 MB genome estimated by the k-mer method in Fig. 3), with an overall G + C content of 47.9%. The genome contains 10,066 predicted open reading frames. The general characteristics of vthe complete genome are summarised in Table 2. The whole-genome sequence data reported in this paper have been deposited in the Genome Warehouse of the BIG Data Center, Beijing Institute of Genomics (BIG), Chinese Academy of Sciences, under accession number GWHAAFV00000000, which is publicly accessible at http://bigd.big.ac.cn/gwh (BIG Data Center Members, 2018). Genomic comparisons between *A. glaucus* ‘CCHA’ and other fungal strains revealed that strain CCHA has a genome size comparable to those of *Magnaporthe grisea* (28.6 MB) (Dean et al., 2005) and *Aspergillus fumigatus* (29.4 MB) (Nierman et al., 2005).

**Table 1:** Summary of the whole-genome shotgun sequencing.

Library ID	Mean size	output (bp)	GC content (%)
11C08038DS4PA_3	543 bp	11,698,985,000	50.0
11C08038P2Q11PB_3	2,195 bp	3,384,228,600	49.9
2W601	15 KB	2,496,238,600	46.2

**Table 2:** Statistics of the genome assembly.

	Scaffolds	Contigs
Counts	76	313
Size	26.0 MB	26.0 MB
Gap_number	214	–
MAX	4.7 Mb	474 KB
N50	795 KB	209 KB
N75	379 KB	–
N90	167 KB	58 KB
Size over 1 MB	6	
GC (%)	47.9	47.9

**Figure 4:**
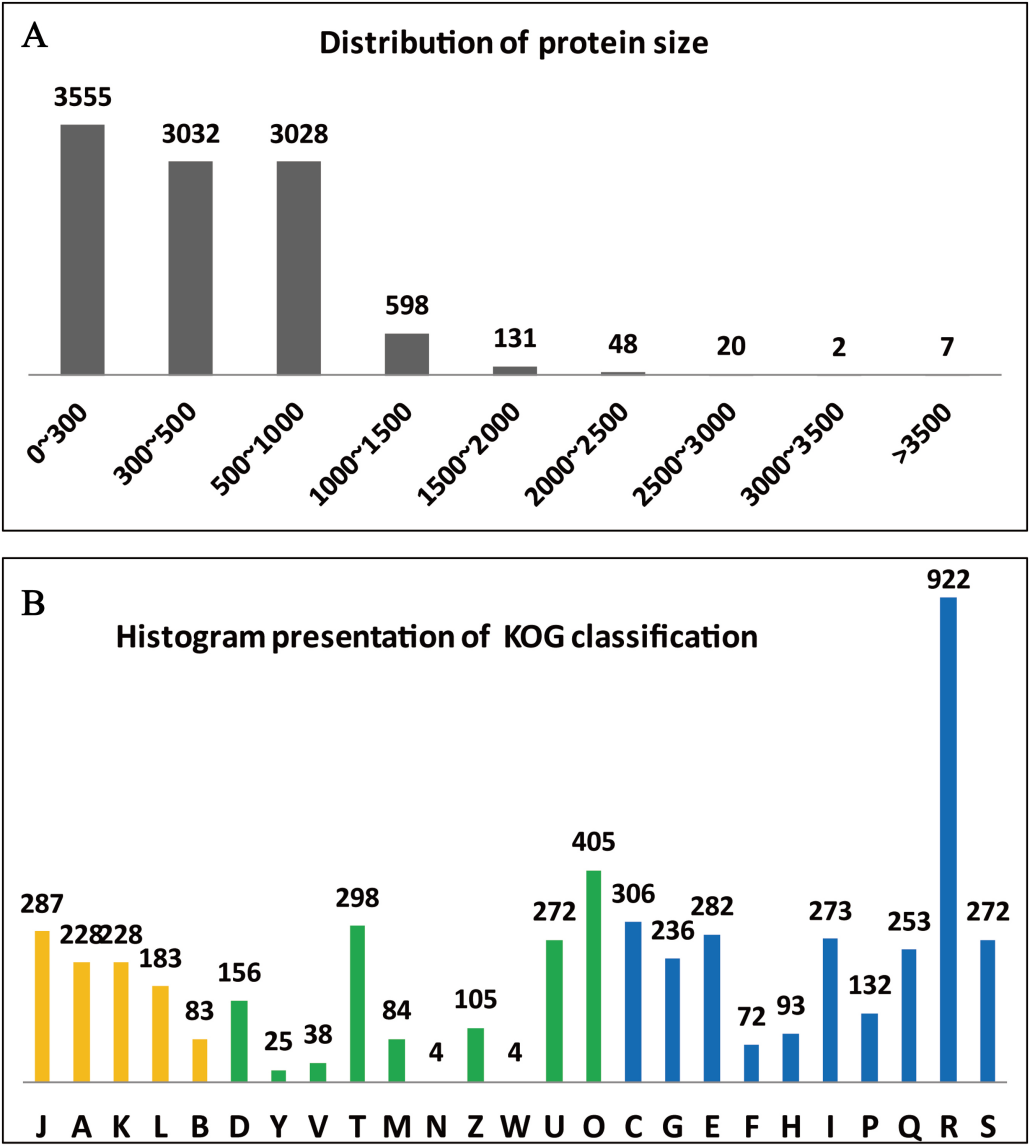
Summary of the genome annotation. (A) Distribution of protein sizes encoded by predicted unigenes. (B) Functional categories of predicted unigenes based on KOG classifications. Yellow bar represents categorical genes for Information storage and processing; green bar for Cellular processes and signalling, and blue for Metabolism. The longitudinal coordinates of the graph A to the Z are as follows: (A) RNA processing and modification, (B) chromatin structure and dynamics, (C) energy production and conversion, (D) cell cycle control, cell division, chromosome partitioning, (E) amino acid transport and metabolism, (F) nucleotide transport and metabolism, (G) carbohydrate transport and metabolism, (H) coenzyme transport and metabolism, (I) lipid transport and metabolism, (J) translation, ribosomal structure and biogenesi, (K) transcription, (L) replication, recombination and repair, (M) cell wall/membrane/envelope biogenesis, (N) cell motility, (O) posttranslational modification, protein turnover, chaperones, (P) inorganic ion transport and metabolism, (Q) secondary metabolism biosynthesis, transport and catabolism, (R) general function prediction only, (S) function unknown, (T) signal transduction mechanisms, (U) intracellular trafficking, secretion and vesicular transport, (V) defence mechanisms, (W) extracellular structures, (Y) nuclear structure, (Z) cytoskeleton.

The clusters of orthologous groups (KOG/COGs) programme is extremely useful for compiling the annotated gene data of a complete genome, for readily describing the data, and for systematically deducing the functions of protein families (Tatusov, Koonin & Lipman, 1997). The predicted 10,066 genes of *A. glaucus* ‘CCHA’ were sorted according to COG classifications (Fig. 4A) and 1,647 of the genes were associated with metabolism, 10.02% with information storage and processing, and 13.82% with cellular processes and signalling. Some of the genes (2.71%) could not be categorised into COG classes because the functions and features of these genes were poorly characterised (Fig. 4B).

### Comparative genome analysis

**Figure 5:**
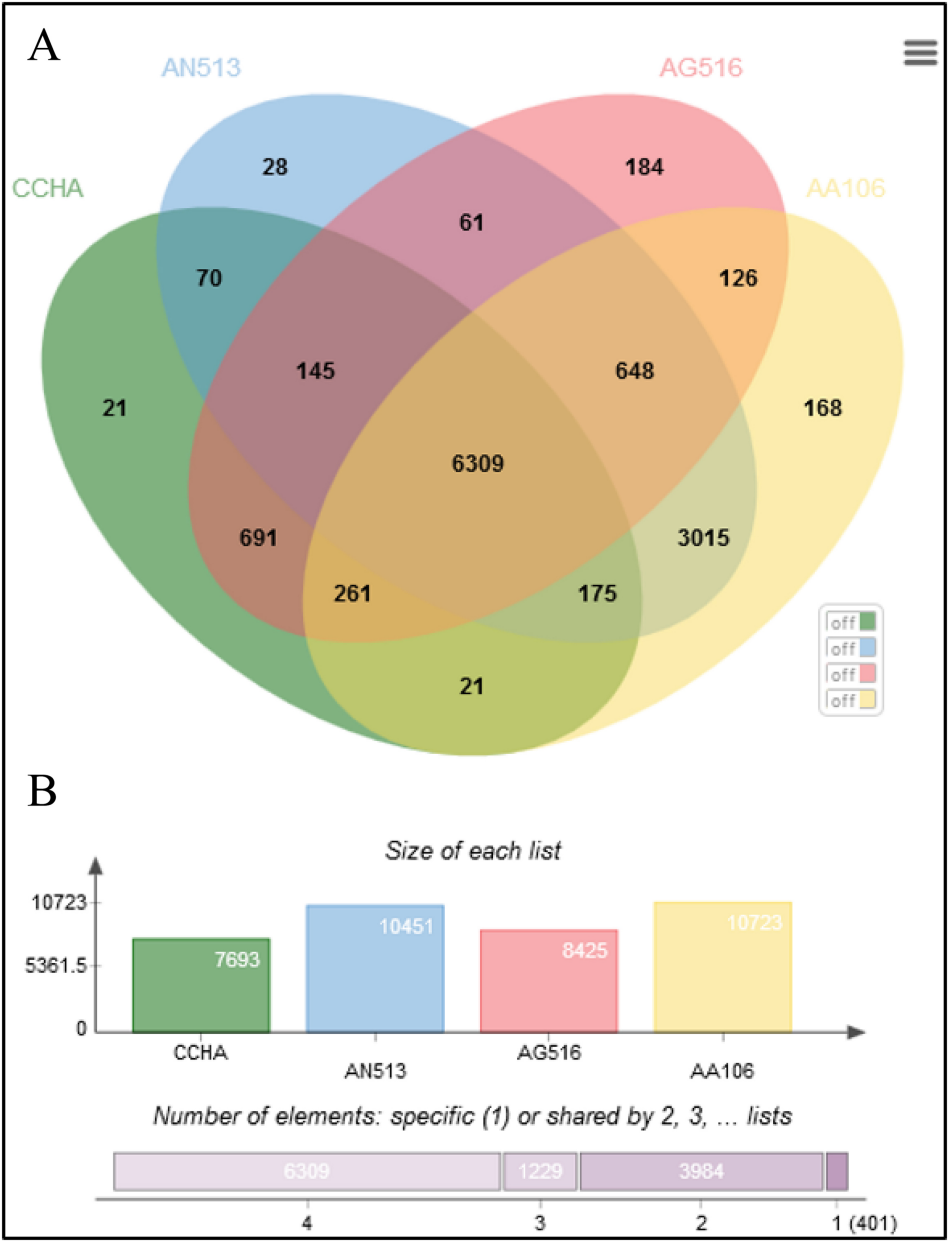
Comparative genome statistics of *A. glaucus* ‘CCHA’ and others. (A) Orthologous group of *A. glaucus*-related species. (B) Venn diagram of unigenes shared by the genomes of closely related *Aspergillus* spp.

The *Aspergillus* strains shared most of the genes assigned to general cellular functions. As shown in the Venn diagram constructed for four representative *Aspergillus* genomes (Fig. 5), all the strains shared 6,309 coding sequences (CDSs). In addition, strain AG516 shared the most (1,079) additional CDSs from the core collection of genes with ‘CCHA’ (Fig. 5A). The fewest unique CDSs (21) were detected in ‘CCHA’, while ‘AG516’ had the greatest number of unique genes.

**Figure 6:**
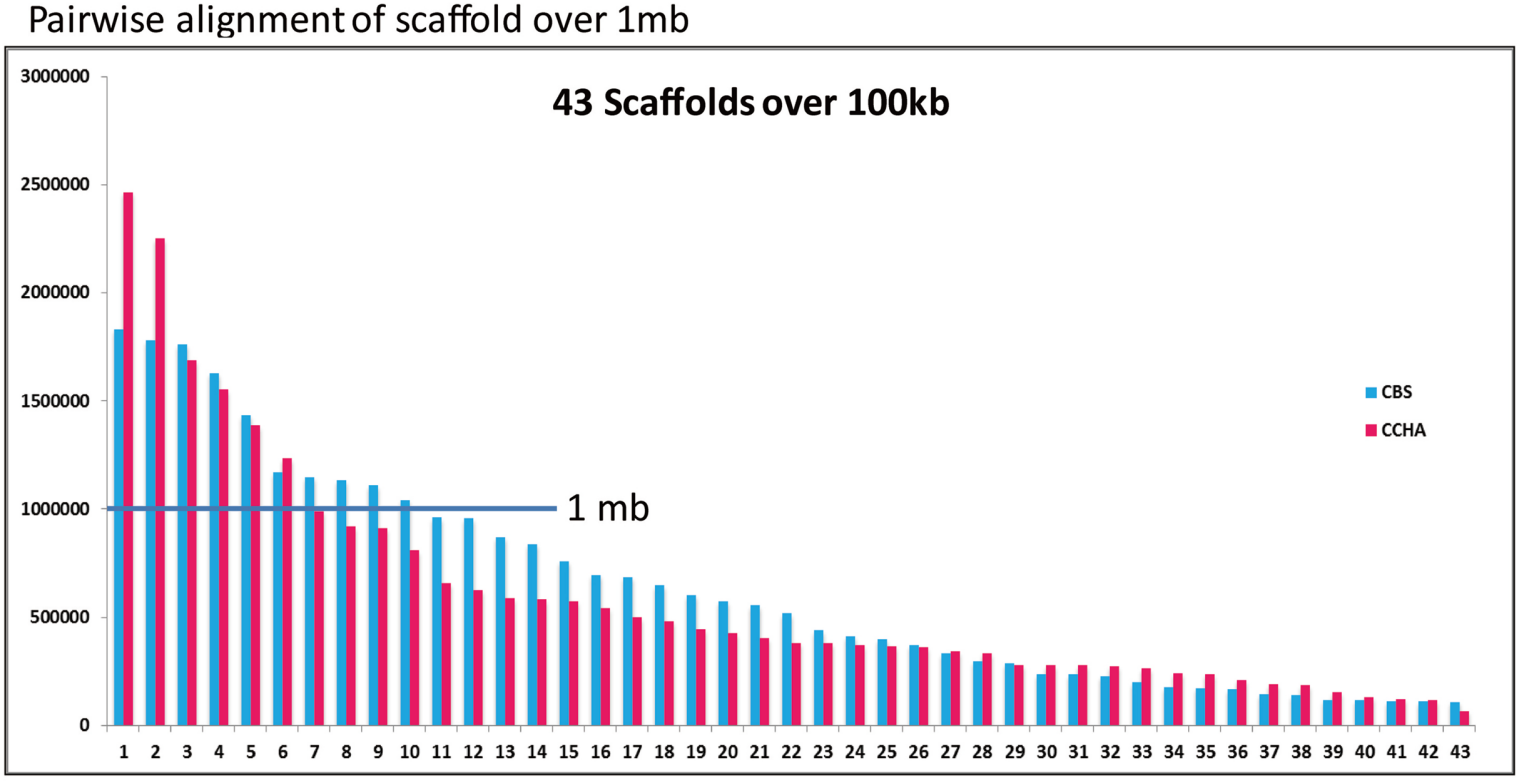
Comparative analysis of *A. glaucus* ‘CCHA’ with ‘CBS’. ****Pairwise alignment of scaffolds in *A. glaucus* ‘CCHA’ with ‘CBS’.

**Figure 7:**
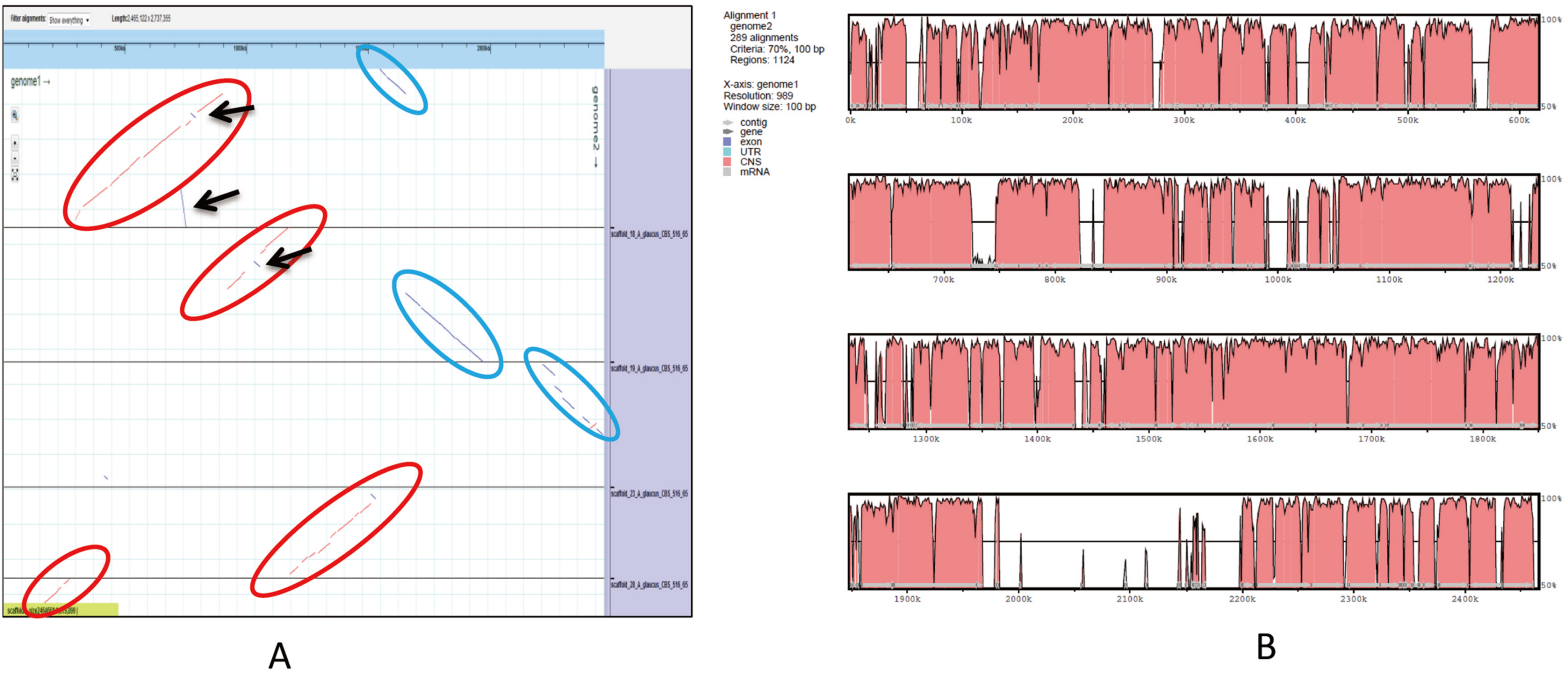
Whole-genome comparison between *A. glaucus* ‘CCHA’ and ‘CBS’. (A) Dot-blot analysis of the genome sequences of *A. glaucus* ‘CCHA’ and ‘CBS’. (B) Global alignment of the genome sequences of *A. glaucus* ‘CCHA’ and ‘CBS’.

An ortholog analysis revealed that the four *Aspergillus* species had 11,923 clusters, 11,522 ortholog clusters that included at least two species, and 5,986 single-copy gene clusters (Fig. 5B). To better understand the relationships among taxa based on the genomes, a MAUVE progressive alignment was performed for ‘CCHA’ with other genomes selected based on the phylogenetic tree of concatenated genes. Strain CBS_516 had a similar gene cluster distribution to that of ‘CCHA’, supporting the assignment of strain CCHA to *A. glaucus* (Fig. 6). To compare the genomes of *A. glaucus* ‘CCHA’ with those of widely recognised *A. glaucus* ‘CBS_516’, a dot-blot analysis was conducted using the RAST programme. The dot-blot analysis showed that the genome of ‘CCHA’ was very similar to that of ‘CBS_516’, as indicated by the diagonal line (Fig. 7A). When the whole-genome sequences were compared using MAUVE software, the locations and sizes of the genes in ‘CCHA’ were similar to those in ‘CBS_516’ (Fig. 7B). Among the genes annotated by a BLAST algorithm-based analysis, conserved genes between ‘CCHA’ and ‘CBS_516’ had high-query coverages.

### Differentially expressed genes

**Figure 8:**
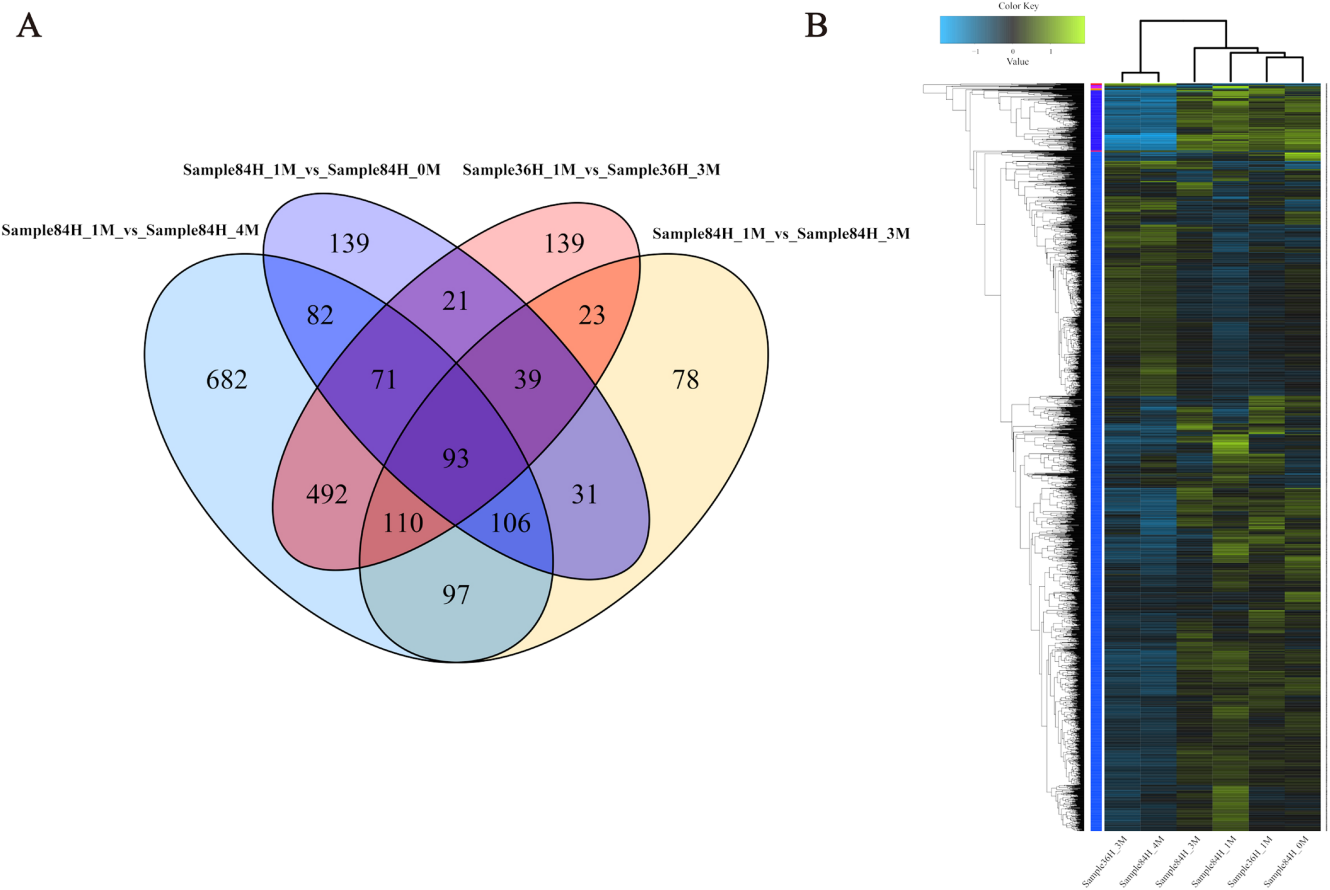
Expression profiles of *A. glaucus* ‘CCHA’ genes under salt stresses. (A) Differentially expressed genes (DEGs) that are unique or shared among different groups. Venn diagram summarising differentially expressed genes in each group. (B) Hierarchical cluster analysis of DEGs. Expression patterns of DEGs after exposure to salt treatments of different concentrations.

To investigate the gene expression profiles and identify the critical genes involved in *A. glaucus* responses to salt stress, we compared the DEGs between different treatment groups (Figs. 8A and 8B; Table 3). There were 889 DEGs in ‘36H’ exposed to a 3-M salt stress compared with ‘36H’ exposed to a 1-M salt stress (Table S1). Here, ion transport-related genes, including sodium P-type ATPase (g4579), voltage-gated K^+^ channel beta subunit (g5829) and MFS transporters were up-regulated in response to salinity. In addition, redox-related oxidoreductases and superoxide dismutases were also enriched. There were 582 DEGs in ‘84H’ exposed to a 0-M salt stress for compared with in ‘84H’ exposed to a 1-M salt stress. Some genes involved in basic metabolism, such as sugar transporters and electron carriers were down-regulated, indicating that the normal development of *A. glaucus* ‘CCHA’ requires a certain amount of salt. Moreover, there were 574 DEGs in ‘84H’ exposed to a 3-M salt stress and 1,731 DEGs in ‘84H’ exposed to a 4-M salt stress compared with those in ‘84H’ exposed to a 1-M salt stress. Long-term stress mainly influenced ion transmembrane transport and redox adjustment-related genes, while under high-salinity stress conditions, secondary metabolism became the driving force behind cellular homeostasis. These results showed that salt stress affects *A. glaucus* ‘CCHA’.

**Table 3:** Sequencing, assembly and differential gene expression statistics for the six transcriptome data.

Sample	Total reads	Clean reads	Mapped reads	Mapping percentage (%)	Gene number		
					Fold > 2	Up	Down
36H_1M	2.42 × 10^7^	2.11 × 10^7^	2.04 × 10^7^	96.51	–	–	–
36H_3M	2.52 × 10^7^	2.19 × 10^7^	2.10 × 10^7^	96.19	839	213	775
84H_0M	2.33 × 10^7^	2.03 × 10^7^	1.92 × 10^7^	95.02	582	234	348
84H_1M	2.43 × 10^7^	2.12 × 10^7^	2.04 × 10^7^	96.54	–	–	–
84H_3M	2.49 × 10^7^	2.16 × 10^7^	2.10 × 10^7^	96.76	577	214	363
84H_4M	2.46 × 10^7^	2.13 × 10^7^	2.07 × 10^7^	97.05	1,733	559	1,174

**Note:**

*A. grucus* ‘CCHA’ grew optimally at 1M salt concentration, so the sample under this condition was chosen as the control. The yellow highlight indicates that the control is Sample36H_1M; for the rest, the control is Sample84H_1M.

### Gene ontology and pathway classification enrichment analyses of DEGs

On the basis of the gene ontology (GO) analysis, an internationally standardised gene functional classification system (Ashburner et al., 2000), DEGs were classified into the three major functional categories of biological process, cellular component, and molecular function (*p*-value < 0.05; Table S2). The GO enrichment analysis based on Sample36h_3M vs. Sample36h_1M showed that in the biological process classification, oxidation–reduction progress, melanin metabolic progress and transmembrane transport were the highly represented categories. For molecular function, oxidoreductase activity was the most highly represented category. Among the DEGs in Sample84h_0M vs. Sample84h_1M, for the biological process classification, oxidation–reduction progress was the most highly represented category. For cellular component, intrinsic component of membrane figured prominently. In Sample84h_3M vs. Sample84h_1M, for the biological process classification, transmembrane transport was the most highly represented category. For molecular function, oxidoreductase activity was the most highly represented category. In Sample84h_4M vs. Sample84h_1M, for the biological process classification, transmembrane transport was the most highly represented category. After comparing these groups, we inferred that the salt condition affected the redox state and transmembrane transport of *A. glaucus*.

To evaluate the functions of DEGs in responses to salt stress, the KEGG database was used to analyse pathways. The results of the KEGG pathway enrichment analysis are shown in Table S3. For Sample36h_3M vs. Sample36h_1M, the DEGs were most highly represented in ‘starch and sucrose metabolism’ and ‘tyrosine metabolism’ pathways (Table S2). For Sample84h_0M vs. Sample84h_1M, the most significant pathways were identified as ‘pentose and glucoronate interconversions’ and ‘starch and sucrose metabolism’. Salt stress had significant effects on multiple pathways in *A. glaucus*, suggesting that these pathways and associated processes might be critical for developmental and metabolic variations in responses to salt stress.

To verify the effectiveness of annotation, a COG classification of up regulated genes for Sample36h_3M vs. Sample36h_1M was performed, resulting in 20 COG groups. Of these, carbohydrate transport and metabolism (10.8%) was the dominant group, followed by translation, ribosomal structure and biogenesis (9.39%) and Amino acid transport and metabolism (8.92%). These results provided multiple candidate genes implicated in responding to high-salt stress in *A. glaucus* ‘CCHA’.

### Isolation and characterisation of salt-tolerance-related genes

**Figure 9:**
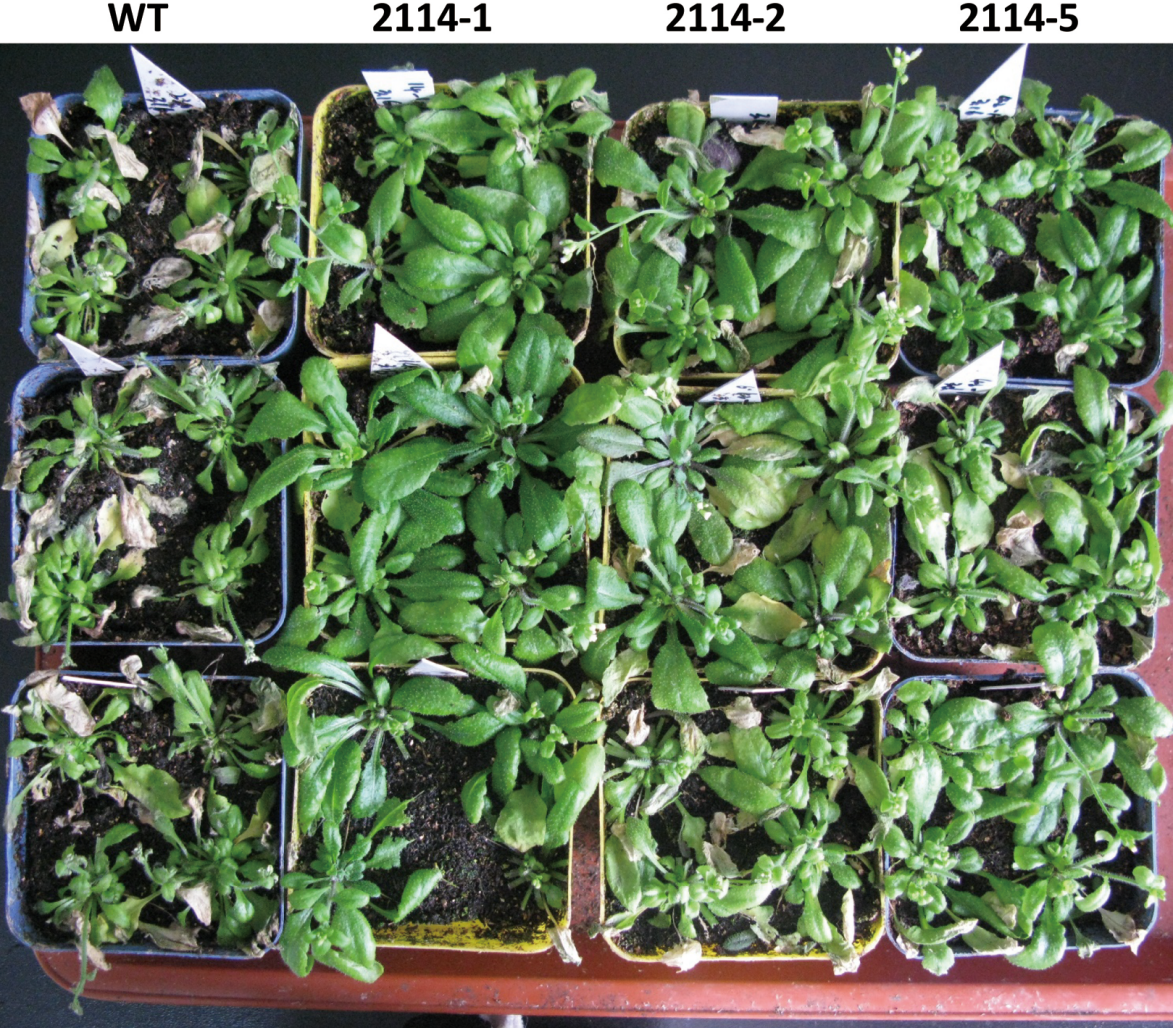
Phenotypes of wild-type (WT) and transgenic *A. thaliana* under long-term salt stresses in the growth chamber. WT (*A. thaliana* of accession Columbia) and transgenic Arabidopsis plants grown under 150 mm NaCl conditions for 9 d were photographed, 2114-1, 2114-2 and 2114-5 mean transgenic lines.

**Figure 10:**
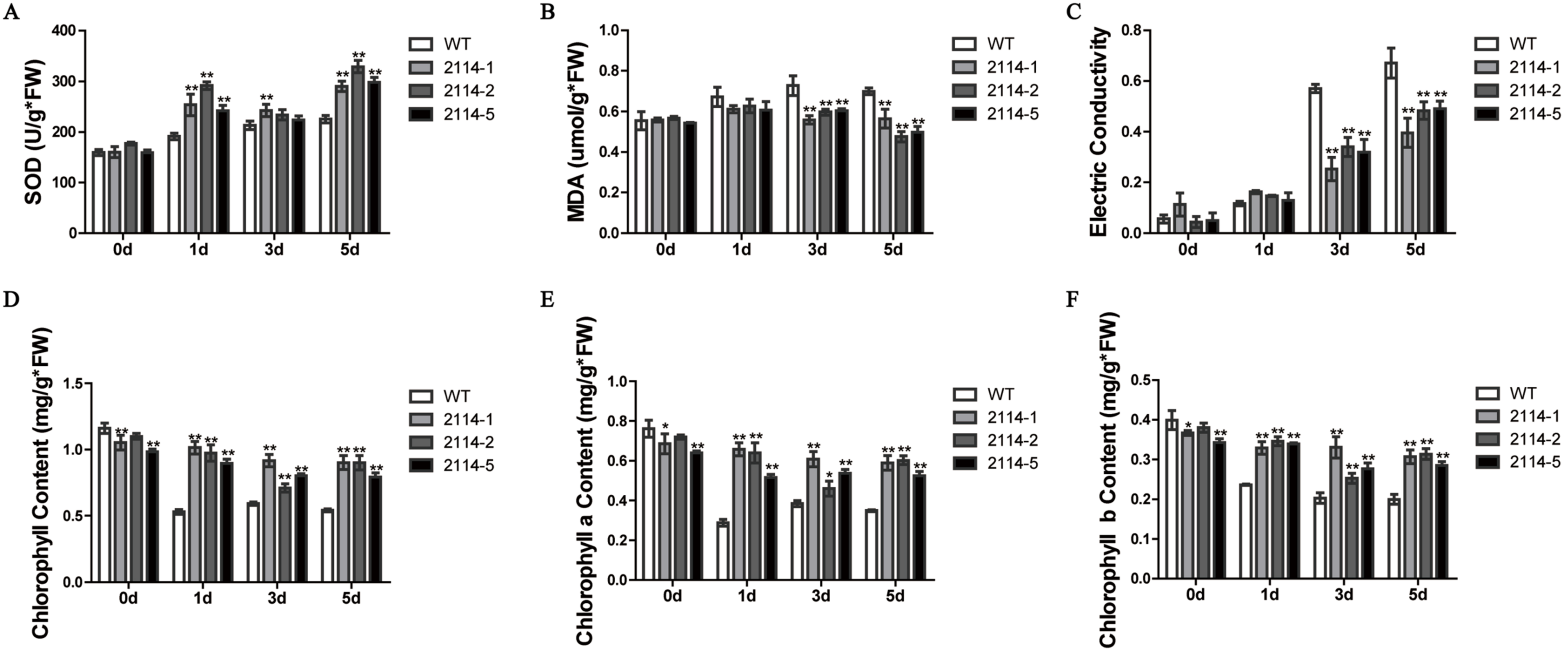
Changes in the SOD and MDA levels, electrical conductivity, and chlorophyll content in the wild-type (WT) and transgenic plants after salt treatments. (A) SOD; (B) MDA levels; (C) electrical conductivity; (D) chlorophyll content; (E) chlorophyll a content; (F) chlorophyll b content. WT means wild type; and 2114-1, 2114-2 and 2114-5 mean transgenic lines. Single or double asterisks indicate a significant difference at *P* < 0.1 or *P* < 0.05 from wild type.

We previously observed that a 0.75-M NaCl treatment caused a severe growth inhibition in *E. coli* ‘BL21’ Star (DE3) cells. Therefore, we hypothesised that one way to identify genes involved in the activation of salt-tolerance in *A. glaucus* ‘CCHA’ was to identify genes that caused growth-inhibition recovery when heterologously expressed in *E. coli* ‘BL21’ (Fang et al., 2014). We hypothesised that mRNAs for tolerance inducers most likely would be expressed highly after exposure to stress and that mRNAs for many regulatory genes would be present at high levels relative to other genes. Consequently, we concluded that the best way to assure randomness in our expression library would be to fuse salt-induced cDNAs to pYES2.0. In this manner, we examined all of the transformants and identified five conditional growth-inhibited recovery mutants (Fang et al., 2014). Then, we verified the salt-stress tolerance of candidate genes in *A. thaliana* using *Agrobacterium*-mediated transformations. *Ag*_*2114*, which encodes a DJ-1/PfpI family protein, confers salt tolerance to *A. thaliana* in transgenic plants (Fig. 9). Three transgenic lines and a WT control line were used for physiological studies under salt-stress conditions. In total, greater damage was observed on the leaves of the WT *A. thaliana* than on the leaves of the transgenic lines when treated with 200 mm NaCl. At all the salt-stress durations, the SOD activity and total chlorophyll, chlorophyll a and chlorophyll b levels, in CCHA-2114-overexpressing plants were significantly greater than those in control plants. Moreover, CCHA-2114’s overexpression led to reductions in the MDA level, lipid peroxidation and ion leakage compared with the untransformed line and thus likely increased the tolerance to salt stress (Figs. 10A–10F).

## Discussion

Salt tolerance is a complex trait involving responses to cellular osmotic and ionic stresses (Zhu, 2000). For microorganisms, the specific habitat’s conditions, such as high salt, hypoxia, or low light intensity, usually results in specific physiological and metabolic behaviour (Bernan et al., 1994; Osterhage et al., 2000). *A. glaucus* ‘CCHA’ is such a strain, even extremely saline conditions, promotes the asexual development of *A. glaucus* (Fig. 1). This trait may render *A. glaucus* ‘CCHA’ a candidate for exploring the response mechanisms to salt stress. To investigate the molecular and genetic principles underlying the adaption of *A. glaucus* ‘CCHA’ to salt stress, we assembled a high-quality reference genome for *A. glaucus* ‘CCHA’ isolated from the surface of wild vegetation growing around a saltern in Jilin, China, based on sequence data from a whole-genome shotgun sequencing platform using Illumina Solexa technology. This assembly contained 106 scaffolds (>1 KB; N50 = ~0.795 MB), has a length of ~26.0 MB and covers ~83% of the predicted genome size (~31.6 MB). In addition to data analyses of comprehensive transcriptomic surveys and comparative genomics, we investigated the molecular mechanisms of *A. glaucus* fungal species’ adaptation to the high-salt environment of the saltern.

Because developmental variations are closely correlated with global life networks, an RNA-seq-based comparative transcriptome analysis was conducted to understand salt stress-driven global life changes in *A. glaucus* ‘CCHA’. Gene expression comparisons among the different treatment groups helped identify candidate genes responsible for the underlying responses to salinity stress in *A. glaucus* ‘CCHA’. Fewer DEGs were found in Sample84h_4M vs. Sample84h_1M (1733) compared with in other groups, which indicated that high salinity may influence more abundant genes. To further unravel the significantly altered biological processes that occur upon salinity stress, the DEGs were subjected to GO and KEGG enrichment analyses. Based on the GO and pathway classifications of the DEGs, the reducing ability and transmembrane transport contributed the most to the salt tolerance of ‘CCHA’. Two major strategies, the ‘osmotic equilibrium’ and the ‘regulation of cellular redox status’ have been adopted by halophiles for salt-stress resistance (Dong et al., 2017; Weinisch et al., 2018) and both are accomplished by strain CCHA. The ‘osmotic equilibrium’ is accomplished through the active movement of Na^+^ into or out of cells to counterbalance its passive diffusion (Zou et al., 2008; Llopis-Torregrosa, Hušeková & Sychrová, 2016). When the environmental osmolality is greater, organisms must compensate for the ion influx. In our study, ion transport-related genes, including *trk1*, were up-regulated in response to salinity stress. *A. glaucus* ‘CCHA’ employed *trk1*, which recycles K^+^ and hyperpolarises the apical membrane, creating a positive cell potential that drives Na^+^ efflux (Calero et al., 2000; Bertl et al., 2003). In addition, DEGs of Sample84h_3M vs. Sample84h_1M were enriched in intrinsic components of membranes, which may indicate that adjusting the membrane permeability is a high-efficiency way to reduce ion diffusion and water content, rather than solely relying on the more energetically demanding mechanisms of ion transport. The ‘regulation of cellular redox status’ process is important in maintaining cellular homeostasis. Under physiological conditions, cells maintain a redox balance through the generation and elimination of reactive oxygen species (ROS) (Rodriguez & Redman, 2005). Normally, redox homeostasis ensures that endogenous and exogenous stimuli respond accurately. However, when redox homeostasis is disturbed, oxidative stress may lead to abnormal development (Ayer, Gourlay & Dawes, 2014). Both exogenous and endogenous sources contribute to the formation of intracellular ROS. Exposure to salinity stress, especially a high salinity stress, activated the redox process in *A. glaucus* ‘CCHA’ to balance the ROS level. Moreover, for the primary metabolism, the KEGG pathway enrichment analysis showed that many DEGs were up-regulated in pathways involved in starch and sucrose metabolism, which might be related to its long-term evolution in a saline environment.

This work increased the understanding of fungal adaptations to salt stress in a saltern environment. It showed that halotolerant fungal development was closely correlated with salt stress. It also indicated that a strong restorative potential and an ion transport ability may be essential reasons for the extreme resistance of halotolerant fungi to salt. The findings may be useful in helping to discover novel candidate genes in fungi that could be used to develop salt-tolerant crops.

## Conclusions

In this study, the genome of *A. glaucus* ‘CCHA’, which was isolated from the surface of plants growing near a salt mine in Jilin, China, was sequenced, and the genes set of this strain were characterised and compared with those of related species. Based on transcriptomic analyses, we determined that the redox state and transmembrane transport might be critical molecular mechanisms for the adaptation of *A. glaucus* ‘CCHA’ to the high-salt environment of the saltern. By expressing a cDNA-library in *E. coli*, we isolated a salt tolerance-related genes *CCHA-2114* and validated its functions in salt stress through heterogeneous expression in *Arabidopsis*, in which it was responsible for promoting SOD activities, maintaining membrane integrity and inhibiting chlorophyll-content decreases. Our work provides a good understanding of *A. glucus* ‘CCHA’s adaption to salt stress and may be useful for mining candidate genes associated with salt tolerance to develop transgenic plants.

## Supplemental Information

10.7717/peerj.8609/supp-1 Supplemental Information 1 Statistics of differential gene expression of *Aspergillus glaucus* ‘CCHA’ in responseto high-salt stress.

10.7717/peerj.8609/supp-2 Supplemental Information 2 GO enrichment of DEGs in this study.

10.7717/peerj.8609/supp-3 Supplemental Information 3 KEGG enrichment of DEGs in this study.

## Abbreviations

DEGs: differentially expressed genes
GO: gene ontology
KOG/COG: clusters of orthologous of proteins
KEGG: Kyoto encyclopedia of genes and genomes

## Additional Information and Declarations

### Data Availability

The following information was supplied regarding data availability: The raw RNA-seq data are available at GenBank: SRX3203744, SRX3203745, SRX3203746, SRX3203747, SRX3203748 and SRX3203749 for *A. glaucus* ‘CCHA’ 36H_1M, 36H_3M, 84H_0M, 84H_1M, 84H_3M and 84H_4M, respectively.
